# Oral Health in Individuals with Severe Mental Illness on Second‐Generation Antipsychotics—A Scoping Review

**DOI:** 10.1111/jop.13639

**Published:** 2025-05-06

**Authors:** Dileep Sharma, Oliver Higgins, Aleksander Sawicki, Poornima Ramamurthy, Belinda Field, Paul Hussein, Kiran Singh, Sarbin Ranjitkar

**Affiliations:** ^1^ Oral Health University of Newcastle Ourimbah New South Wales Australia; ^2^ Healthy Minds Hunter Medical Research Institute Lambton Heights New South Wales Australia; ^3^ College of Medicine and Dentistry James Cook University Smithfield Queensland Australia; ^4^ Mental Health Central Coast Local Health District Wyong New South Wales Australia; ^5^ Oral Health Service Central Coast Local Health District Wyong New South Wales Australia; ^6^ Eleanor Duncan Aboriginal Services Mardi New South Wales Australia; ^7^ Adelaide Dental School University of Adelaide Adelaide South Australia Australia

**Keywords:** antipsychotics, dental caries, periodontal disease, sialorrhea, side effects, xerostomia

## Abstract

**Introduction:**

There has been a significant increase in antipsychotic usage, particularly belonging to second‐generation antipsychotics (SGAs), in the management of severe mental illnesses (SMIs) over the past few decades, but their impact on oral health is unclear. This review evaluated the oral side effects of the SGAs used in managing SMIs.

**Methods:**

A PRISMA‐guided scoping review was conducted, using predefined criteria and relevant word combination in Medline (PubMed), Embase, Scopus and Cochrane databases. Eligible studies were assessed for study type, population demographics, antipsychotic medication usage, oral diseases and conditions, and their outcomes. Primary outcomes were explicitly studied oral conditions or diseases, and secondary outcomes were broader oral or general “treatment‐emergent adverse effects.”

**Results:**

Twelve studies met the inclusion criteria for the primary outcomes and 13 for the secondary outcomes. The most reported primary outcomes were salivary gland dysfunction (hypofunction, three studies; hypersalivation, two studies), dental caries (positive association, three studies; no association, one study), and periodontal disease (five studies). Secondary outcomes included dysgeusia and oral hypoesthesia (six studies).

**Discussion:**

SGAs significantly compromise oral health, particularly through salivary gland and sensory dysfunction, highlighting the need for up‐to‐date guidelines on routine oral screening and preventive care.

## 1 Introduction

Severe mental illness (SMI) includes conditions such as psychotic disorders, schizophrenia, bipolar disorder, and major depressive disorder with psychotic features or treatment‐resistant depression [1, 2, 3, 4]. It often encompasses severe forms of anxiety disorders, eating disorders, and personality disorders. These conditions can significantly affect quality of life due to recurrent psychiatric hospitalizations and coexisting substance abuse, leading to social issues such as homelessness, unemployment, and incarceration [3]. Management primarily involves pharmacological treatment with antipsychotics, mood stabilizers, anti‐anxiety and anti‐depressants, and other psychotropic medications [4].

Antipsychotics, also known as neuroleptics, are a major class of medications used to manage SMIs. They are generally classified into different generations based on their characteristics and mechanisms of action. Introduced in the 1950s, the first‐generation “typical” antipsychotics (FGAs), including chlorpromazine and haloperidol, primarily block dopamine receptors in the mesolimbic pathway in the brain [5]. Although therapeutically effective in reducing psychosis, they are often associated with severe side effects including movement disorders (extrapyramidal symptoms and tardive dyskinesia). Developed in the 1980s to address these drawbacks, the second‐generation “atypical” antipsychotics, including risperidone, clozapine, olanzapine, and quetiapine, target multiple neurotransmitters (e.g., serotonin and dopamine) in different regions of the brain [5]. Second‐generation antipsychotics have shown improved efficacy in treating both positive and negative symptoms of psychosis while causing fewer movement disorders [6]. However, they carry an increased risk of adverse effects, including weight gain, diabetes, hypertension, hematological disorders, and cardiometabolic complications [7, 8]. Third‐generation antipsychotics, introduced in the early 2000s, aimed to further improve treatment outcomes, for example, aripiprazole is a partial dopamine agonist that helps to optimise dopamine levels [6].

People with SMIs, particularly schizophrenia, have historically been known to experience greater prevalence of dental caries, periodontal disease, oral infections, and salivary dysfunction [9, 10]. A 2017 review summarized the oral side effects of several FGAs and SGAs, including salivary secretion dysfunction, from three databases: The Monthly Index of Medical Specialties (MIMS), UpToDate, and Meyler's Side Effects of Drugs Encyclopaedia [11]. While these databases serve as concise prescribing guides, they lack in‐depth and evidence‐based critical analysis. A landmark systematic review on medication‐induced salivary gland dysfunction remains a key evidence‐based resource [12, 13]; however, it requires updating to incorporate more recent research findings.

FGAs have been in clinical use longer than SGAs and have accumulated a greater volume of research, highlighting the need for more evidence synthesis for other classes of antipsychotics. Hence, this scoping review was conducted to collate current evidence on oral diseases and conditions associated with SGAs in individuals diagnosed with SMI.

## 2 Methodology

This scoping review was conducted using the framework described by Aarskey and O'Malley, and the Joanna Briggs Institute [14, 15, 16]. The population, intervention, comparator, outcome, and study characteristics (PICOS) framework was used to formulate a focused research question (Table 1).

**TABLE 1:** PICOS framework for structure.

Population	Intervention	Context	Outcomes	Study characteristics	
				Inclusion	Exclusion
Any gender; any age group; humans with exposure to antipsychotic medication in an in‐patient or out‐patient setting	Second‐generation antipsychotics	Severe mental illness	Oral and dental disorders or conditions	Peer‐reviewed studies; prospective or retrospective observational (cross‐sectional or cohort) studies; randomized controlled trials from 1996 onwards	Grey literature; animal or in vitro studies; all types of reviews, studies prior to 1996; non‐English studies; no full‐text availability

### 2.1 Research Question

The following question was addressed: What are the oral diseases and conditions in individuals second‐generation antipsychotics for severe mental illnesses? The primary outcomes were explicitly studied oral conditions or diseases, and secondary outcomes were broader oral or general “treatment‐emergent adverse effects.”

### 2.2 Search Strategy

Primary studies were identified through Medline via PubMed, Embase, Scopus, and Cochrane databases. The PRISMA‐ScR (Preferred Reporting Items for Systematic reviews and Meta‐Analyses extension for Scoping Reviews) guidelines were followed for conducting and reporting, with a PRISMA flow chart used to visually depict the literature search [17, 18].

The search strategy included peer‐reviewed articles published in English between 2000 and 2024. The key concepts for the Medline search via PubMed were: SMI, antipsychotic medications, and dental or oral disorders (Table 2). The search terms, developed and executed by K.S. and D.S., were verified by two university librarians. Synonyms were added for each concept based on preliminary searches of key articles for relevant terminology. Thus, a logic grid was created with key concepts and corresponding MeSH terms. Minor changes were made to comply with search rules and requirements of the Embase and Scopus databases. Search results were finalized by February 23, 2025, and exported to EndNote 20.

**TABLE 2:** Database search terms used for Medline (PubMed).

Key concepts	Search terms
Antipsychotic medications	(Antipsychotic*[tw] OR psychopharmaceutical*[tw] OR Psychotropic*[tw] OR Psychotropic drug*[tw] OR Psychoactive drug*[tw] OR Neuroleptic*[tw] OR valproate[tw] OR Valproic acid[tw] OR clozapine[tw] OR inpatient[tw] OR in patient[tw] OR out patient[tw] OR outpatient[tw] OR treatment*[tw] OR “Antipsychotic agents”[mh] OR “Psychotropic drugs”[mh] OR “Valproic acid”[mh] OR “Clozapine”[mh] OR “inpatients”[mh] OR “outpatients”[mh] OR “therapeutics”[mh] OR “Atypical Antipsychotics”[mh] OR “Second Generation”[mh])
Dental disorders	(periodontal disease*[tw] OR Periodontitis[tw] OR Periapical periodontitis[tw] OR Oral disease*[tw] OR Mouth Disease[tw] OR Oral micro*[tw] OR Stomatognathic disease*[tw] OR Periapical abscess*[tw] OR Periodontal abscess*[tw] OR Dental abscess*[tw] OR Oral dysbiosis[tw] OR Caries[tw] glossodynia*[tw] OR burning mouth syndrome*[tw] OR oral ulceration*[tw] OR orofacial pain*[tw] OR tooth grinding*[tw] OR bruxism*[tw] OR dysgeusia*[tw] OR altered taste*[tw] OR hypoesthesia*[tw] OR oral numbness*[tw] OR Temporomandibular joint disorders*[tw] OR oral trauma*[tw] OR oral self injury*[tw] OR “Periodontal diseases”[mh] OR Periodontitis[mh] OR “Periapical periodontitis”[mh] OR “Mouth diseases”[mh] OR “Stomatognathic diseases”[mh] OR “periapical abscess”[mh] OR “Periodontal abscess”[mh] OR “Dental caries”[mh])
Severe mental illness	(Bipolar disorder*[tw] OR Severe mental illness*[tw] OR Neuroinflammation[tw] OR schizophrenia*[tw] OR mania*[tw] OR dissociative disorder*[tw] OR Psychosis[tw] OR psychoses[tw] OR psychotic[tw] OR psychotic disorder*[tw] OR Severe mental disorder*[tw] OR “Mental disorders”[mh] OR “schizophrenia”[mh] OR “mania”[mh] OR “dissociative disorders”[mh] OR “psychotic disorders”[mh] OR “mental disorders”[mh])

A data extraction template was drafted by the principal investigator to determine relevant variables and was reviewed by the research team. Data were subsequently extracted for administrative details (i.e., author name and year of publication), study parameters (location and study duration), and characteristics (sample size, age, gender, medication, and oral diseases). Saliva flow reduction was recorded as xerostomia (self‐reported) and hyposalivation (clinically‐tested), while increased flow was recorded as drooling (self‐reported) and hypersalivation or sialorrhea (clinically‐tested). A retrospective cohort study for the primary outcomes had two papers, 3 years apart, reporting different outcomes (dental caries and periodontal disease) [19, 20]. Although two other studies for secondary outcomes were related to the same sample pool, they assessed different outcomes, with a 3‐week randomized controlled trial (RCT) [21] to a 50‐week open label, flexible dosage trial [22]. These were treated as separate sources while ensuring no data duplication.

## 3 Results

**FIGURE 1:**
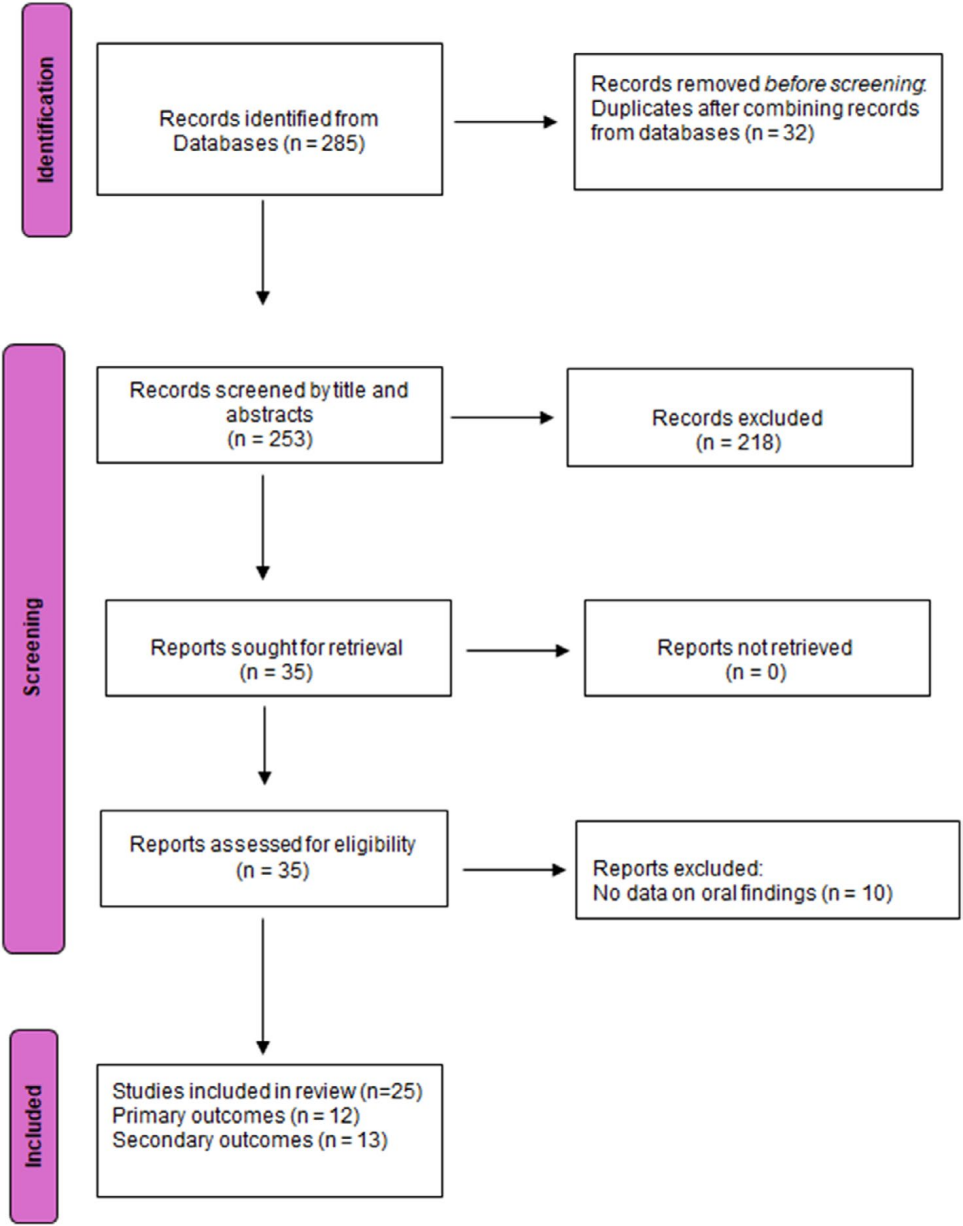
The screening process followed in the scoping review using PRISMA guidelines.

The PRISMA flow chart is shown in Figure 1. Initial searches through the databases yielded 285 articles, from which 32 duplicates were removed. Two reviewers (K.S. and D.S.) screened the titles and abstracts, excluding 218 articles that did not meet the inclusion criteria (Table 1). They independently screened the full texts of the selected articles for primary outcomes, while S.R. and D.S. screened for secondary outcomes, resolving inconsistencies with a third reviewer (P.R.). For the final review, 12 articles met the criteria for primary outcomes (explicitly investigated oral conditions or diseases) and 13 for secondary outcomes (oral conditions identified as “treatment‐emergent adverse effects”).

Table 3 presents study characteristics and primary outcomes. More than half of the studies were cross‐sectional (712), along with three retrospective cohort studies [19, 20, 30], one prospective cohort study [28], and one RCT (Table 3) [26]. They were conducted predominantly within a single country, including Egypt [30], Hungary [28], Israel [32], Japan [26, 31], Norway [25], Serbia [27], Taiwan [19, 20, 29], and Turkey [24], with one study involving multiple Organization for Economic Co‐operation and Development (OECD) countries [23]. The pooled sample comprised 6260 participants, with half (six) studies involving SGA monotherapy [23, 24, 26, 29, 30, 32] and the other half involving polydrug use [19, 20, 25, 27, 28, 31]. Four studies also compared the oral effects of FGAs and SGAs [19, 20, 31, 32]. Most participants had a schizophrenia diagnosis, with one study also including other single or multiple mental disorders [25].

**TABLE 3:** Study characteristics and primary outcomes of the papers included in the scoping review, stratified by oral conditions or diseases.

Authors	Study type	Country	Study population and demographics	Control group/comparison	Antipsychotics reported	Oral diseases or conditions	Outcomes
Tandon et al. 2020 [23]	Cross‐sectional, web‐based survey; *N* = 435 outpatients	United States, Canada, Australia, Spain, Italy, Norway, Denmark	435 patients with stable SCZ taking SGAs for 1–12 months; age 37.9 ± 11 years; M 46.2%, F 53.8%	—	SGA monotherapy	Quality of Life Enjoyment and Satisfaction Questionnaire Short Form (Q‐LES‐Q‐SF), and the Glasgow Antipsychotic Side‐Effect Scale (GASS), including general and oral side effects (e.g., xerostomia, drooling, and dysgeusia, etc.)	Xerostomia 63.9%; drooling 3.7%; Xerostomia was the top third side effect of SGAs. Multiple side effects significantly compromised daily functioning and quality of life and satisfaction
Eltas et al. 2013 [24]	Cross‐sectional study; *N* = 53 outpatients	Turkey	53 schizophrenia (SCZ) patients on SGAs for ≤ 2 years; age 30.5 ± 9.5 years; M 46.5%, F 53.5%	No specific control group; comparison: xerostomia‐inducing SGAs (33) versus sialorrhea‐inducing SGAs (20)	Xerostomia‐inducing SGAs (risperidone, quetiapine, and olanapine 62%) versus sialorrhea‐inducing SGA (clozapine 38%)	Hyposalivation, sialorrhea, DMFT, periodontal disease (plaque index, BOP, probing depth, attachment levels)	Xerostomia‐inducing > sialorrhea‐inducing SGAs for plaque index (80% vs. 58%) and BOP (73% vs. 49%). Clozapine > xerostomia‐inducing SGAs for salivary flow rate (0.23 ± 0.25 vs. 1.12 ± 0.94 mL/min). Similar measures for DMFT data and all other periodontal disease indicators
Frigaard et al. 2023 [25]	Cross‐sectional study; *N* = 46 inpatients (short‐term)	Norway	23 pychiatric patients with single or multiple disorders (including psychosis, personality disorders, and bipolar disorder); 73.9% on SGAs; age 36.2 ± 13.2 years; M 35%, F 65%	23 age‐ and gender‐matched control group (university recruits)	SGAs (89.5%) of all antipsychotics ±20 other types of medications (e.g., antidepressants, sedatives, opiods, anti‐diabetics, and bisphosphonate, etc.)	Xerostomia (Summated Xerostomia Inventory (SXI) questionnaire), Clinical Oral Dryness Score (CODS), caries (DMFT, DT, MT, FT), gingivitis, periodontitis, halitosis, OHIP‐14 (quality of life) score	Psychiatric patients had significantly worse oral health and poorer quality of life than the control group; higher levels of xerostomia (10.4 ± 2.7 vs. 6.0 ± 0.7), oral dryness (5.1 ± 1.9 vs. 0.6 ± 1.2), gingivitis (31% vs. 0%), halitosis (48% vs. 4%), dysgeusia (43% vs. 4%), and OHIP‐14 score (17.9 ± 12.7 vs. 1.04 ± 1.9); no significant differences in DMFT or its components; patients had 29% generalized or localized periodontitis versus presumably 0% for control group
Ishigooka et al. 2018 [26]	Randomized, double‐blind, placebo‐controlled trial; *N* = 459 inpatients	Japan	343 patients with acute SCZ; age 44.3 ± 11.7 years; M 47.5%, F 52.5%	116 placebo patients	SGA (brexpiprazole; 1, 2, or 4 mg/day)	Treatment‐emergent adverse events, including dental caries (no method of assessment)	Brexpiprazole improved SCZ symptoms but increased dental caries for SGA use (5.2% for 1 mg; 0.0% for 2 mg; 3.5% for 4 mg; 0.9% in placebo)
Djordjevic et al. 2020 [27]	Cross‐sectional study; *N* = 140 inpatients	Serbia	70 patients with SCZ or other mental disorders, mainly on SGAs; age 18.9 ± 3.1 years; M 48%, F 52%	70 control patients	SGAs monotherapy or combination [SGAs (90%)^a^, FGAs (28%)^a^, and anxiolytics (70%)^a^]	Caries (DMFT, DT, MT, FT)	SCZ patients (mainly on SGAs) had higher DMFT scores and higher DT scores (4.3 ± 2.7 vs. 0.6 ± 0.9)
Aghasizadeh Sherbaf et al. 2024 [28]	Prospective case–control study; *N* = 100 inpatients and outpatients	Hungary	50 SCZ patients in remission; age: 51.86 ± 13.28 years; M 58%, F 42%	50 control patients	SGAs (e.g., olanzapine, risperidone), benzodiazepines, mood stabilizers	Caries (DMFT, DT, MT, FT, and DMFS); periodontal disease: (plaque index, PI; bleeding on probing, BOP; pocket depth, PD; and attachment loss, AL)	SCZ was significantly associated with poorer oral health (caries and periodontal disease), exacerbated by poorer lifestyle factors (smoking and alcohol); caries: higher DT (8.2 ± 5.2 vs. 4.2 ± 4.2); periodontal disease: higher PI (57.0 ± 23.2 vs. 27.4 ± 17.5), higher BOP% (59.0 ± 22.9 vs. 23.6 ± 17.6), deeper PD (2.8 ± 0.7 vs. 2.2 ± 0.5), and greater AL (3.4 ± 1.7 vs. 2.5 ± 0.8)
Chu et al. 2016 [29]	Cross‐sectional study; *N* = 878 inpatients	Taiwan	878 SCZ patients; age 8.4 ± 5.9 years; M 74%, F 26%	—	SGAs (59.3%) (specific medications not specified)	Caries experience (DMFT with no DT, MT, or FT data)	SGA use was not significantly associated with DMFT
Hu et al. 2016 [19]	Retrospective cohort study; *N* = 3610 outpatients	Taiwan	3610 newly diagnosed SCZ patients; age: 34.7 ± 14.3 years; M 52.4%, F 47.6%	—	FGA (83.2%)^a^, SGA (73.8%)^a^; anticholinergics, and antihypertensives	Caries incidence from treatment records; no direct salivary assessment but dental caries incidence was used as a proxy for saliva flow	Significant increase in caries risk for FGAs (adjusted OR 2.04) but not for SGAs (adjusted OR 1.16)
Shalaby et al. 2023 [30]	Retrospective cohort study; *N* = 64 outpatients	Egypt	42 SCZ patients taking prolactin‐inducing or sparing SGAs; age 40.6% ≤ 50 years, 59.4% ≥ 50 years; M 67.2%, F 32.8%	22 newly diagnosed SCZ patients receiving no treatment	Prolactin‐inducing SGAs for 1 year (e.g., risperidone) versus prolactin‐sparing SGAs for 1 year (e.g., clozapine, quetiapine, and aripiprazole) versus no treatment	Periodontal disease (pocket depth, PD; clinical attachment loss, AL; gingival recession; tooth mobility; and bleeding on probing (BOP)); bone density and serum prolactin levels	Prolactin‐inducing > prolactin‐sparing antipsychotic or no treatment for PD, AL, and serum prolactin levels. Bone mineral density was significantly lower for both antipsychotic groups than no treatment. All antipsychotics potentially contribute to periodontal disease
Okamoto et al. 2016 [31]	Cross‐sectional study; *N* = 127 outpatients	Japan	127 SCZ patients (medication period 10.9 ± 5.3 years); age 57.2 ± 12.8 years; M 44%, F 56%	No specific control group; comparison: FGAs versus SGAs	FGAs (44%)^a^, SGAs (56%)^a^; anxiolytics, antiparkinson drugs, and laxatives	Oral moisture meter to measure hyposalivation objectively	Hyposalivation was associated with xerostomia (subjective assessment); FGAs > SGAs for hyposalivation
Grinshpoon et al., 2015 [32]	Cross‐sectional study; *N* = 348 inpatients	Israel	348 patients hospitalized for SCZ; age 51.4 ± 14.5 years; M 69%, F 31%	No specific control group; *comparison: FGAs* versus *SGAs*	SGAs (11.5%) versus FGAs (46.8%) versus both (41.6%)^a^	Caries (DMFT, DT, MT, FT)	FGAs > SGAs for DMFT scores (23.5 ± 9.9 vs. 19.0 ± 10.5), no significant difference in DT scores (2.3 ± 3.4 vs. 3.4 ± 5.0)
Hu et al. 2019 [20]	Retrospective cohort study (from 2‐year treatment records); *N* = 3610 outpatients	Taiwan	3610 newly diagnosed SCZ patients; 65.7% had treatment for periodontal disease	No specific control group; comparison: FGAs versus SGAs	FGA (83.2%)^a^, SGA (73.8%)^a^	Periodontal disease	Periodontal disease risk was significantly associated with both antipsychotics; FGAs (OR 1.89) > SGAs (OR 1.33)

Abbreviations: DMFT, Decayed, Missing, and Filled Teeth index; DT, decayed teeth; MT, missing teeth; FT, filled teeth; FGA, first‐generation antipsychotic; OHIP, Oral Health Impact Profile‐14; OR, odds ratio; SGA, second‐generation antipsychotic.

^a^ Concomitant prescriptions (SGA polydrug therapy).

Primary outcomes were reported for SGA use with salivary gland dysfunction, dental caries, and periodontal disease. Three studies reported significant associations between SGA use (mono‐ or polytherapy) and reduced saliva flow (one on hyposalivation [24] and two on xerostomia [23, 25]). Interestingly, two studies found significant associations between SGA monotherapy and increased salivary flow; one reported drooling without specifying medications [23], while another reported sialorrhea with clozapine and hyposalivation with risperidone, quetiapine, and olanzapine [24]. Two studies assessing the Decayed, Missing, or Filled Teeth (DMFT) index lacked carious tooth data [24, 29], making their findings inconclusive for caries prevalence. Three studies—an RCT (SGA monotherapy) [26], a prospective case–control study (SGA polytherapy) [28], and a cross‐sectional study (SGA polytherapy) [27], found significant associations with dental caries, while another cross‐sectional (SGA polytherapy) did not [25]. A retrospective cohort study of 3610 newly diagnosed individuals with schizophrenia found no significant association between SGA polytherapy and caries‐related conditions (including tooth wear), but the data are inconclusive for caries assessment. All five studies—one prospective case–control (SGA polytherapy), two retrospective cohort (SGA mono‐ and polytherapy), and two cross‐sectional (SGA mono‐ and polytherapy)—reported significant associations between SGA use and periodontal disease [20, 24, 25, 28, 30]. One of those retrospective cohort studies [30] found a higher prevalence of periodontal disease after a year of treatment with prolactin‐inducing SGAs (amisulpride, risperidone, and paliperidone) compared with prolactin‐sparing SGAs (clozapine, quetiapine, olanzapine, ziprasidone, and aripiprazole) or no treatment. In that study, osteoporotic conditions (low bone mineral density) were greater in prolactin‐inducing SGAs (57.1% osteoporosis, 19.0% osteopenia) and prolactin‐sparing SGAs (19.1% osteoporosis, 47.6% osteopenia) compared to no treatment (18.2% osteoporosis, 4.5% osteopenia). Additionally, two studies showed higher prevalences of xerostomia [31] and periodontal disease [20] for FGAs than SGAs, but no difference for dental caries [20, 32].

Table S1 presents study characteristics and secondary outcomes. Ten studies were RCTs [21, 22, 33, 34, 35, 36, 37, 38, 39, 40], whereas three were cross‐sectional [41, 42, 43]. Three studies on olanzapine and quetiapine polytherapy reported xerostomia as a side effect [33, 35], whereas two on quetiapine and asenapine polytherapy did not [36, 38]. An asenapine monotherapy study also reported hypersalivation [37]. All six studies on sublingual asenapine mono‐ or polytherapy for managing clozapine‐induced sialorrhea reported oral hypoesthesia (numbness) [38, 40] or combined dysgeusia (bitter taste) and oral hypoesthesia [21, 22, 37, 39]. Temporomandibular disorder (TMD), including bruxism, was highly prevalent in patients with schizophrenia and other psychiatric conditions (78.9% for SGA polytherapy for TMD [43]; 58.5% for SGA monotherapy for bruxism [41]). Furthermore, TMD symptoms were more severe with FGAs than SGAs [43] or worsened with combined FGA‐SGA polytherapy compared to either alone [41]. Burning sensations in the buccal mucosa and tongue affected around 45% of bipolar disorder patients withon FGA or SGA monotherapy, with no difference between medication types [42].

## 4 Discussion

SGAs are gaining popularity for the management of SMIs [6], but they can produce various adverse effects, traditionally categorized as activating (restlessness, jittery feeling, sleeplessness), sedating (dizziness, sleepiness, difficulty thinking or concentrating), endocrine (sexual dysfunction) and metabolic effects (weight gain, glucose intolerance, dyslipidaemia, and hypertension), all of which can significantly compromise the quality of life [7, 23, 44, 45, 46]. This was highlighted in a worldwide, web‐based survey assessing the quality of life using the Quality of Life Enjoyment and Satisfaction Questionnaire Short Form (Q‐LES‐Q‐SF) and the Glasgow Antipsychotic Side‐Effect Scale (GASS) [23]. Among the reported side effects, xerostomia was a significant factor compromising quality of life [23]. Salivary gland dysfunction, often presenting as xerostomia, is a major side effect of many medications, including FGAs, certain SGAs, anxiolytics, antidepressants, cannabinoids, and mood stabilizers [47, 48].

Salivary gland dysfunction was primarily assessed subjectively (using the Summated Xerostomia Inventory questionnaire [25] or targeted questions [23]), with few objective measures (using the Clinical Oral Dryness Score [25], salivary flow rate [24], or an oral moisture meter [31]) or a combination of both [25]. All four included studies on primary outcomes attributed reduced saliva flow to SGA use, supported by 3/5 studies on secondary outcomes. This is crucial, as reduced saliva flow due to anticholinergic side effects diminishes oral cleansing and buffering, increasing the risks of halitosis, dental caries, periodontal disease, and oral infections (e.g., candida infections due to reduced histatin 5 levels) [49, 50, 51, 52].

Sialorrhea is a dose‐dependent side effect of certain SGAs, often persisting post‐discontinuation, primarily with clozapine but occasionally with ‘xerostomia‐inducing’ SGAs (e.g., risperidone and ariprazole) [53, 54, 55, 56]. Clozapine has been linked with parotitis and painful salivary gland swelling [57, 58], while sialorrhea can disrupt laryngeal peristalsis and suppress swallowing reflex [59] and pose physical and psychosocial problems, including dysphagia, dehydration, aspiration pneumonia, foul odor, angular cheilitis, and social stigma [60, 61, 62]. Although the pathophysiology of hypersalivation remains unclear, clozapine‐induced hypersalivation is considered paradoxical due to its potent α2‐ adrenergic antagonistic, high M4‐muscarinic, and anticholinergic effects [53, 63, 64]. Antipsychotics often exhibit dual effects—muscarinic (hypersalivatory) and anticholinergic (hyposalivatory)—which may explain occasional hyposalivation with “sialorrhea‐inducing SGAs” and hypersalivation with ‘xerostomia‐inducing’ SGAs [12]. The epigenetic mechanisms regulating these salivary shifts remain unclear. Additionally, no direct research has investigated whether sialorrhea has a protective effect against certain oral diseases, including dental caries.

Dental caries was reported to be strongly associated with SMI or substance use, in a recent umbrella review of 11 meta‐analyses [9]; however, no data were provided on medication types. Increased caries risk was also associated with SGAs in most included studies in the current review. This can be attributed to medication‐induced hyposalivation combined with other behavioral factors, such as inability to self‐care, low motivation, poor communication, dental phobia, financial constraints, substance use, and poor oral hygiene practices [9, 65, 66, 67, 68].

Periodontal disease was consistently reported for SGAs by all five included studies in the current review, including a large retrospective study that attributed a higher risk to FGAs than SGAs [20]. A recent review has suggested a possible link between oral microbial dysbiosis, including the role of phageoma (bacteriophages), and neuroinflammation in schizophrenia [69]. Drug‐induced hyposalivation can indirectly increase periodontal disease activity through the plaque accumulation and microbial alteration in favor of gram‐negative anaerobic bacteria [70]. Significantly higher prevalence of salivary *Porphyromonas gingivalis* has also been identified to be a risk factor for periodontal disease in schizophrenia [71]. Additionally, all antipsychotics have the potential to reduce bone density and increase the susceptibility to periodontal disease, but ‘prolactin‐inducing’ antipsychotics have a greater propensity for alveolar bone resorption [30].

Six clinical studies on secondary outcomes, primarily involving RCTs, reported oral dysgesia and hypoesthesia as side effects of sublingually administered asinapine, an SGA alternative used to manage clozapine‐induced sialorrhea. Antipsychotic‐induced metabolic disorders, for example, diabetes, can impair immune function and wound healing, while clozapine‐associated agranulocytosis (a severe form of neutropenia), though rarer than previously thought, can lead to life‐threatening infections [72]. Bruxism is a common occurrence in individuals with SMI, yet one included study found no significant association to SGA use [41], while another reported a lower prevalence for SGAs than FGAs [43]. Another uncommon risk of SGAs and other antipsychotics is tardive dyskinesia, including abnormal involuntary orofacial movements (e.g., tongue thrusting, lip smacking, and dysphagia) [73]. There is a paucity of data on orofacial tardive dyskinesia associated with SGAs, and most existing literature on oral dyskinesia is based on older studies. Although multiple studies, including meta‐analyses, have reported a reduced TD risk with SGAs than FGAs [74], their negative impact on oral hygiene and increased risk of plaque‐associated oral conditions cannot be overlooked [75].

There are established guidelines for the use of specific antipsychotics, including clozapine for treatment‐resistant psychotic disorders (e.g., schizophrenia), emphasizing the need to balance effectiveness, side effects, and individual preference in patient‐centered care [76, 77]. Due to the risks of metabolic syndrome, hypertension, hematological effects, and weight gain, mandatory monitoring of clozapine is required in some countries, including Australia and the United Kingdom [8, 78]. Periodic general health screening is required, with prompt referral as necessary [8]. Monitoring visits are recommended either weekly (for ≤ 18 weeks on clozapine) or monthly (> 18 weeks and stable dosage) to rule out clozapine‐induced agranulocytosis.

This is the first scoping review presenting updated evidence on SGA‐related oral side effects that are relevant to general dental practitioners, special needs dentists, oral medicine specialists, and allied health professionals. One of its key strengths is identifying gaps and challenges that will help to direct future research and collaborative opportunities; however, it also has limitations. No studies have directly compared the risks and benefits of taking antipsychotics versus none, presumably due to ethical challenges, making it challenging to assess the long‐term effects of SGAs on oral health. The review also has the inherent limitation of being unable to assess the risk of bias. It excluded low‐level evidence, such as a case report of olanzapine‐induced black hairy tongue resolving after dose reduction, which could still inform clinical practice in the absence of more robust data [79].

There was a wide heterogeneity in the included studies, with the sample sizes varying widely from 46 to 3610 participants per study [19, 20, 25]. Many included studies reported SGA polytherapy (primary outcomes, 50%; secondary outcomes, 53.8%) combined with other psychotropic drugs, potentially exacerbating side effects. Most studies excluded individuals with complex comorbidities and serious mental conditions (including suicide ideation), limiting applicability to these cases. Most studies were observational (case control and cross‐sectional), with only a handful of RCTs enabling assessment of causal relationships. Lastly, the measure of oral conditions varied widely; for example, one study reporting caries prevalence along with other general side effects lacked methodological details [26]. Other studies using only the DMFT index or broad “caries‐related diagnoses” (including tooth wear) were deemed inconclusive.

Given the potential serious side effects of antipsychotics, a key question arises: is an individual better or worse off with them? Philosophically, harm may outweigh benefits if symptoms are mild, manageable without medication (through behavioral approaches), and the medication causes serious side effects. On the other hand, benefits will outweigh risks if the medication effectively manages severe symptoms, prevents relapses, and reduces the risk of serious deterioration, self‐harm, or harm to others. These pertinent issues require further evaluation through a multiprofessional approach.

## 5 Conclusions and Recommendations

Targeted routine oral health assessments, such as frequent dental visits and preventive care, are recommended for individuals with SMI, including those on SGAs. Patient education, oral hygiene maintenance, and modification of lifestyle factors are important for addressing the underlying causes. Preventive and minimally invasive management should prioritize salivary dysfunction, dental caries, periodontal disease, oral infection, dysgeusia, oral hypoesthesia, orofacial pain, and temporomandibular disorders. General dental practitioners can typically manage routine dental conditions, while soft tissue conditions, orofacial pain, and complex cases may require oral medicine or special needs specialists.

Interprofessional collaboration among mental health practitioners, primary care providers, and dental professionals is essential for comprehensive, patient‐centered care addressing both mental and oral health. Serious medication side effects may require dose adjustments or alternative treatments. Xerostomia management includes lifestyle modification (e.g., increased hydration, reducing caffeine and alcohol, and avoiding smoking) and oral care products (e.g., saliva substitutes, sialogogues, sugar‐free and nonacidic chewing gum, remineralizing products, and alcohol‐free mouthwash). Medication‐induced sialadenosis (salivary gland hypertrophy) may require medication adjustment, infection prevention, and, in severe cases, botulinum toxin injections or surgical gland reduction. Preventive caries management involves dietary modification (e.g., limiting sugary or acidic foods), while periodontal disease prevention could require oral hygiene and systemic health management. Addressing underlying systemic conditions, such as Sjogren's syndrome, diabetes, and agranulocytosis, is crucial. Management of temporomandibular disorders should follow standard protocols, including nightguard to prevent tooth wear and fracture when indicated.

Further controlled studies are needed to improve the quality of evidence on the long‐term oral effects of specific SGAs and other antipsychotics. Interprofessional collaboration should be encouraged to monitor and updating guidelines that support evidence‐based prescribing and promote oral and general health.

## Author Contributions

Conceptualization: D.S., O.H., and P.R. Data curation: D.S., O.H., P.R., K.S., and S.R. Methodology: D.S., O.H., B.F., P.H., P.R., and S.R. Formal analysis: D.S., O.H., P.R., K.S., and S.R. Resources: D.S., O.H., and A.S. Data curation: D.S., O.H., P.R., K.S., and S.R. Visualization: D.S., O.H., and S.R. Supervision: D.S., O.H., and S.R. Project administration: D.S. and O.H. Funding acquisition: D.S. Writing – original draft preparation: D.S., O.H., P.R., K.S., A.S., P.H., B.F., and S.R. Writing – review and editing: D.S., O.H., and S.R. All the authors contributed to writing and revising the draft. All the authors approved the final version of this manuscript.

## Conflicts of Interest

The authors declare no conflicts of interest.

## Supporting information


**Table S1.** Study characteristics and secondary outcomes of the papers included in the scoping review, stratified by antipsychotics used and adverse oral effects.

## Data Availability

Data sharing is not applicable to this article as no new data were created or analyzed in this study.
