# Supplementary material for: Oral Health in Individuals with Severe Mental Illness on Second‐Generation Antipsychotics—A Scoping Review
Source: J Oral Pathol Med. 2025 May 6;54(6):401–12. doi: 10.1111/jop.13639 (PMC12230892; doi:10.1111/jop.13639)
Supplement: Supplementary file 1 — Table S1. Study characteristics and secondary outcomes of the papers included in the scoping review, stratified by antipsychotics used and adverse oral effects. [file JOP-54-401-s001.docx]

Supplementary Table 1: Study characteristics and secondary outcomes of the papers including in the scoping review, stratified by antipsychotics used and adverse oral effects

| **Author(s)** | **Study type** | **Country** | **Study population and demographics** | **Control group/ comparison** | **Antipsychotic used** | **Treatment-emergent oral adverse effects** |
| --- | --- | --- | --- | --- | --- | --- |
| Brown et al., 2005 [33] | Randomized, double-blind 7-week trial; N = 410 outpatients | USA | 205 patients with bipolar I disorder and depression; Age 37.0±11.1 y; M 40%, F 60%) | 205 patients on lamotrigine (anticonvulsant) | Olanzapine (SGA) /Fluoxetine (SSRI) Combination (OFC) vs. Laotrigine | OFC use was associated with **xerostomia** |
| Sachs et al., 2004 [34] | Randomized, double-blind, placebo-controlled 3-week trial; 105 inpatients | USA | 56 patients with acute bipolar mania: Baseline mean age 40.5 y; M 57%, F 44%) | 49 patients on placebo + lithium/divalproex | Quetiapine + lithium/divalproex | Most common side effects in ≥10% patients included **xerostomia** (18.9% vs. 4.0%) |
| Yatham et al., 2005 [35] | Randomized, double-blind, placebo-controlled study for 3 to 6 weeks; N = 402 inpatients for 1 week | Unclear but likely Canada, Europe, South Africa, Sweden, and USA | 197 patients with bipolar I disorder; Mean age around 39-40 y (range 18-70y); M 53%, F 47% | 205 patients on placebo + lithium/divalproex | Quetiapine + lithium/divalproex | Quetiapine **increased xerostomia** (19.4% vs. 3.0%) |
| Vieta et al., 2008 [36] | Randomized controlled trial for up to 104 weeks; N = 754 outpatients | Multicentre, parallel study in USA, Australia, Europe and South Africa | 336 patients with bipolar I disorder; Age 42.1±12.7y; M 45%, F 55% | 337 patients on placebo + lithium/divalproex | Quetiapine twice daily + lithium/divalproex | Quetiapine - **no xerostomia** (no further data provided) |
| Dogterom et al., 2009 [37] | Study 1: Placebo-controlled, double-blind, randomised, parallel-group, multiple-dose study; Study 2: open-label, multiple-ascending-dose study; 70 inpatients | USA, Netherlands | 30 pediatric patients with SCZ or bipolar I disorder; Age 10-17 y; M 58%, F 42% | 40 placebo patients | Asenapine (1-10 mg twice daily) | 5/70 experienced **severe dysgesia and oral hypoesthesia**; **1/70 experienced mild/moderate hypersalivation** |
| Findling et al., 2015 [21] | Randomised, double-blind, placebo-controlled 3-week trial; N = 403 inpatients | USA, Russia | 302 pediatric patients with bipolar I disorder; Age 13.8±2.0 y; M 47%, F 53%) | 101 placebo patients | Asenapine (2.5, 5, or 10 mg twice daily) | Combined **dysgesia, oral hypoesthesia,** (20.2-25.3% vs. 4.0%) |
| Findling et al., 2016 [22] | 50-Week Open-Label, Flexible-Dose Trial (at the completion of a 3-week trial (Findling et al., 2015); N = 321 inpatients | USA, Russia | 241 pediatric patients with bipolar I disorder; Age 13.8±2.0 y; M 50%, F 50%) | 80 placebo patients; comparison: asenapine (3 weeks)/asenapine (50 weeks) vs. placebo (3 weeks)/asenapine (50 weeks) | Asenapine (2.5-10 mg twice daily) | Asenapine/asenapine < placebo/asenapine for TCAs (combined **dysgeusia and oral hypoesthesia**, 3.7% vs 18.8%) |
| Szegedi et al., 2012 [38] | Randomized, double-blind study (12-week core + 40-week extension); N = 401 inpatients (likely) | Australia, India, Korea, Russia, Thailand, USA | 199 patients with bipolar I disorder + acute mixed mood episodes; Mean age ~39y, SD ~12y; M 57%, F 43% | 202 control patients on lithium/valporate only | Asenapine (5 - 10 mg twice daily) + lithium/valproate | **Oral hypoesthesia** (6.0% vs. 1.0%); **no significant for xerostomia** (3.5% vs. 4.0* |
| Landbloom et al., 2010 [39] | Randomised, double-blind, placebo-controlled 6-week trial; 311 inpatients | USA, Canada, Russia, Bulgaria, Croatia, Ukraine | 201 patients with acute SCZ; Age 40.6±11.2 y; M 58%, F 42% | 101 placebo patients on 46 on olanzapine; Comparison: asenapine vs. olanzapine vs. placebo | Asenapine (2.5 mg and 5.0 mg twice daily vs. Olanzapine (15.0 mg once daily) vs. placebo | Combined **dysgeusia and oral hypoesthesia** incidence: 5.0 mg asenapine (7.1%) > 2.5 mg asenapine (5.2%) > olanzapine (0.0%) or placebo (0.0%) |
| Mahajan et al., 2013 [40] | Parallel group, open label, randomized controlled trial; N = 120 inpatients | India | 60 patients with acute mania; Mean age around 36 y (SD around 11y; M 38%, F 62% | Comparison: 60 patients on olanzapine + divalproex | (Asenapine + divalproex) vs. (olanzapine + divalproex) | **Tongue hypoesthesia** occurred only with asenapine (36.0%) |
| Namli et al., 2024 [41] | Cross-sectional study; 211 inpatients | Turkey | 124 SCZ patients with probable bruxism; Age 51.0±9.3 y; M 53%, F 48% | 87 patients without probable bruxism; comparison: FGAs + SGAs vs. FGAs vs. SGAs | FGAs and SGAs | A high proportion of patients (58.5%) had probable bruxism; **no significant effect of SGA and bruxism**; c**ombined FGAs + SGAs** > **FGAs or SGAs for probable bruxism** |
| Meurman and Murtomaa, 2024 [42] | Cross-sectional questionnaire & interview study; N = 102 inpatients | Finland | 102 psychiatric patients; Mean age ~37-40 y; M < F | 170 patients with SMIs. No specific control group; comparison FGAs (59%) vs. SGAs (41%) | FGAs vs. SGAs | FGAs = SGAs for **oral pain** (45.0% vs. 43.0%), but **FGAs > SGAs for xerostomia** (66% vs. 53%, respectively) |
| Oflezer et al., 2020 [43] | Cross-sectional study; 222 inpatients | Turkey | 112 patients with bipolar disorder and probable bruxism; Age 34.5±9.6 y; M 44%, F 56%) | 110 patients with no probable bruxism | SGAs + mood stabilizers (MSs), MSs, FGAs, depot antipsychotics, antidepressants | (SGAs + mood stabilizers) > other treatments for reducing **probable bruxism** |

FGA, first generation antipsychotic; SGA, second generation antipsychotic

SGAs assessed generally were more effective in managing mental conditions than control or placebo medications

General side effects are not presented here, but some are serious including a suspected suicide in one study*
